# Multiscale Entropy of the Heart Rate Variability for the Prediction of an Ischemic Stroke in Patients with Permanent Atrial Fibrillation

**DOI:** 10.1371/journal.pone.0137144

**Published:** 2015-09-01

**Authors:** Eiichi Watanabe, Ken Kiyono, Junichiro Hayano, Yoshiharu Yamamoto, Joji Inamasu, Mayumi Yamamoto, Tomohide Ichikawa, Yoshihiro Sobue, Masehide Harada, Yukio Ozaki

**Affiliations:** 1 Department of Cardiology, Fujita Health University School of Medicine, Toyoake, Japan; 2 Division of Bioengineering, Graduate School of Engineering Science, Osaka University, Toyonaka, Japan; 3 Department of Medical Education, Nagoya City University Graduate School of Medical Sciences, Nagoya, Japan; 4 Educational Physiology Laboratory, Graduate School of Education, University of Tokyo, Tokyo, Japan; 5 Department of Neurosurgery, Fujita Health University School of Medicine, Toyoake, Japan; Scuola Superiore Sant'Anna, ITALY

## Abstract

**Background:**

Atrial fibrillation (AF) is a significant risk factor for ischemic strokes, and making a robust risk stratification scheme would be important. Few studies have examined whether nonlinear dynamics of the heart rate could predict ischemic strokes in AF. We examined whether a novel complexity measurement of the heart rate variability called multiscale entropy (MSE) was a useful risk stratification measure of ischemic strokes in patients with permanent AF.

**Methods and Results:**

We examined 173 consecutive patients (age 69±11 years) with permanent AF who underwent 24-hour Holter electrocardiography from April 2005 to December 2006. We assessed several frequency ranges of the MSE and CHA_2_DS_2_-VASc score (1 point for congestive heart failure, hypertension, diabetes, vascular disease, an age 65 to 74 years, and a female sex and 2 points for an age≥75 years and a stroke or transient ischemic attack). We found 22 (13%) incident ischemic strokes during a mean follow up of 3.8-years. The average value of the MSE in the very-low frequency subrange (90–300 s, MeanEn_VLF2_) was significantly higher in patients who developed ischemic strokes than in those who did not (0.68±0.15 vs. 0.60±0.14, P<0.01). There was no significant difference in the C-statistic between the CHA_2_DS_2_-VASc score and MeanEn_VLF2_ (0.56; 95% confidence interval, 0.43–0.69 vs. 0.66; 95% confidence interval, 0.53–0.79). After an adjustment for the age, CHA_2_DS_2_-VASc score, and antithrombotic agent, a Cox hazard regression model revealed that the MeanEn_VLF2_ was an independent predictor of an ischemic stroke (hazard ratio per 1-SD increment, 1.80; 95% confidence interval, 1.17–2.07, P<0.01).

**Conclusion:**

The MeanEn_VLF2_ in 24-hour Holter electrocardiography is a useful risk stratification measure of ischemic strokes during the long-term follow-up in patients with permanent AF.

## Introduction

Atrial fibrillation (AF) is an important risk factor for ischemic strokes, making the prevention of ischemic strokes with oral anticoagulants a mainstay of the current AF clinical practice [1, 2]. To aid in the decisions for prophylaxis and to reduce the exposure of low-risk patients to bleeding complications, a robust risk stratification scheme of the stroke likelihood would be of considerable practical value. To date, as a thromboembolic risk stratification scheme, the CHA_2_DS_2_-VASc score (Congestive Heart failure, hypertension, Age≥75 years [doubled], Diabetes, Stroke [doubled], Vascular disease, Age 65–74 years, and Sex category [female sex]) has been established to guide antithrombotic therapy in individuals with AF [3, 4].

During AF, rapid and random atrial impulses create disorganized atrioventricular (AV) nodal conduction and, thus, generate irregular fluctuation in the ventricular response interval (VRI). Such conditions seem to preclude the application of a standard heart rate variability (HRV) analysis [5]. Recently, novel mathematical analyses of the HRV have been developed that provide fresh insight into the abnormalities of the heart rate behavior [6–9], and its association with cardiovascular events [10]. Of these, the multiscale entropy (MSE) analysis was first described by Costa et al. [8, 9] as a method for analyzing the complexity of nonlinear and nonstationary signals in finite length time series. For instance, an MSE analysis allows for the characterization of complex temporal fluctuations inherent in permanent AF [8, 9], as well as provides additional prognostic information in a clinical setting [11]. To date no studies have examined whether the MSE analysis could predict the ischemic strokes in AF patients. We therefore examined whether the MSE represented an independent risk of ischemic strokes in permanent AF patients and compared the predictive value of ischemic strokes with the CHA_2_DS_2_-VASc score.

## Methods

### Patients

We studied consecutive permanent AF patients at our hospital that underwent 24-hour Holter electrocardiograms (ECGs) from April 2005 to December 2006 recorded either as outpatients or during hospitalization. Permanent AF was defined as AF of >1 year of duration, in patients with no evidence of intervening sinus rhythm and in whom there was no plan to restore sinus rhythm [2]. We excluded patients with complete AV block, sustained ventricular tachycardia, ventricular ectopy >5% of the 24-hour total beats, cardiac pacemakers, paroxysmal AF, valvular AF or prosthetic heart valves, taking rhythm control drugs, or who had a 24-hour Holter ECG recording in which periods of artifact or noise representing >5% of the total monitoring time occurred. Patients who had acute coronary syndrome, strokes or hemodynamic instability in the preceding 6 months, as well as any surgical interventions during the same period, were also excluded. The CHA_2_DS_2_-VASc score was recorded as a baseline measurement of the stroke risk [3]. The study was approved by the ethics committee of Fujita Health University and conformed to the principles outlined in the Declaration of Helsinki. All patients provided their written informed consent at the time of the Holter recording.

### Holter monitoring and the measurements of the heart rate variability and irregularity

The Holter ECG was recorded with a 2-channel digital recorder (Fukuda Denshi, Tokyo) during the patient’s usual daily activities. Patients were asked to submit their diary symptoms and daily activities. ECG signals were digitized at 125 Hz and 12 bits and processed offline using a personal computer equipped with dedicated software. Only recordings with at least 22 hours of data were included in the analysis. The results of the automatic analysis were reviewed, and any errors in the R-wave detection and QRS labeling were edited manually.

We determined the following time domain measures of the variability of the VRI; SD of all the R-R intervals (SDVRI), SD of the 5-minute averages (SDAVRI) to quantify the magnitude of the deviation from the mean, and frequency domain measures including the ultra-low frequency (ULF: <0.0033 Hz), very-low frequency (VLF: 0.0033–0.04 Hz), low frequency (LF: 0.04–0.15 Hz), and high frequency (HF: 0.15–0.4 Hz) bands. As a measure of the irregularity of the VRI, we employed an MSE analysis [8, 9, 12]. The MSE analysis was comprised of two steps: 1) coarse-graining (local averaging) of the observed time series into different time scales; and 2) quantification of the degree of regularity in each coarse-grained time series using a sample entropy [7]. In this study, the sample entropy was calculated using a pattern length *m* of 2 and a similarity factor *r* of 0.15, and the analyzed VRI time series were interpolated with a linear interpolation and resampled at 2 Hz. To quantify the MSE profile in a plot of the sample entropy vs. frequency (log_10_ scale), we calculated the mean value of the sample entropy (MeanEn) and the linear-fitted slope of each scale range corresponding to the frequency-domain measures, namely, the HF (2.5–6.5 s), LF (6.5–25 s), and VLF (25–300 s). In addition, the VLF range was further divided into two subranges, the VLF1 (25–90 s) and VLF2 (90–300 s), respectively. Note that unlike the previous studies [8, 11], the time scale unit of the MSE profile was not in beats but in seconds because the analyses based on the beat scale could be affected by the cardiac rhythm (sinus or non-sinus) and heart rate. In addition, the frequency range of the HRV, such as the HF, LF and VLF, have been defined based on characteristic time scales involved with heart rate modulation. Therefore, we calculated the MSE based on the time scale and compared the MSE measures with the conventional frequency-domain measures. To examine the association between the MSE measures and cardiac autonomic activity, we further determined the degree of concealed conduction of the AV node estimated according to a technique called the Lorenz plot technique (scattering index) [13–15].

### Follow-up and endpoint

Members of the events verification committee who were blinded to the clinical background reviewed the medical records. Valid ischemic strokes were defined as neurological deficits, of sudden onset, that persisted for more than 24 h, and that were not explained by other etiologies (e.g., trauma, infection, or vasculitis). The diagnosis of the ischemic strokes was made by a neurosurgeon either by the use of computed tomography or magnetic resonance imaging. Ischemic strokes included both those due to cardiac emboli and atherothrombotic infarctions.

### Statistical analysis

Differences between the two groups were evaluated using a Student’s t-test for continuous variables and the χ^2^ test or Fisher’s exact probability test for categorical data. The relationship between the quantitative variables was assessed by a Spearman’s rank correlation analysis. A multiple regression analysis was used to test the relationship between clinical variables and MSE. Receiver-operating characteristic curve (ROC) analyses were generated to test the predictive discrimination of significant VRI parameters to identify the association with ischemic strokes during the follow-up. We used Cox models to determine the association of the HRV parameters with the time to an ischemic stroke and we adjusted it for the age, CHA_2_DS_2_-VASc score and the use of antithrombotic agents (warfarin or antiplatelet drug). The hazard ratio (HR) and 95% confidence interval (CI) are given. The cumulative probabilities of an ischemic stroke were estimated as a function of the time using the Nelson-Aalen method and comparisons between the groups were performed using a log-rank test. Training and testing were conducted with a cross-validation technique [16]. The significance of diurnal variation in the MeanEn_VLF2_ was tested using a one-way analysis of variance (ANOVA) and Bonferroni post-hoc test. Quantitative data are expressed as the mean±standard deviation (SD) and categorical variables as a percentage. A two-tailed P-value of <0.05 was considered significant. The statistical analyses were performed with JMP 10.0.2 software (SAS Institute, USA) and the R statistical package (www.r-project.org).

## Results

During the recruitment period, 202 consecutive patients were assessed for enrollment eligibility. Twenty-nine patients were excluded according to predetermined clinical criteria.

### Patient characteristics

The baseline clinical characteristics of the 173 patients classified according to the occurrence of ischemic strokes are summarized in Table 1. The mean age of all patients was 69±11 years old (range 30 to 89), of which 35% were ≥75 years old, and 71% were male. There was no significant difference in the CHA_2_DS_2_-VASc score between the 2 groups. Warfarin was being taken by 93 (54%) patients and 30 of those patients were receiving antiplatelets as well. Warfarin was being taken by 88 (53%) in whom the CHA_2_DS_2_-VASc score was ≥1.

**Table 1:** Baseline clinical characteristics of the patients.

	Ischemic stroke (n = 22)	No ischemic stroke (n = 151)	P-value
Age, years	71±8	69±11	0.35
Female, n (%)	7 (32)	43 (28)	0.26
Underlying disease, n (%)			
Congestive heart failure	9 (41)	56 (37)	0.72
Hypertension	15(68)	88 (58)	0.38
Diabetes	1(5)	14 (9)	0.46
Stroke or TIA	9 (41)	40 (26)	0.16
Vascular disease	2 (9)	15 (10)	0.90
CHA_2_DS_2_-VASc score	3.3±1.6	3.2±1.5	0.38
Echocardiographic findings			
Left atrial dimension, mm	45±9	46±11	0.81
Ejection fraction, %	56±9	54±10	0.61
Medications, n (%)			
Beta-blocker	4 (18)	24 (16)	0.79
Digitalis	8 (36)	65 (43)	0.55
Ca-channel blocker	7 (32)	35 (23)	0.38
ACE inhibitor	7 (32)	37 (25)	0.47
Diuretics	9 (41)	56 (37)	0.75
Antiplatelet	5 (23)	41 (27)	0.66
Warfarin	10 (45)	83 (55)	0.40

Ischemic stroke: patients who developed a stroke during the follow-up, No ischemic stroke: patients who remained ischemic stroke free during the follow-up. CHA_2_DS_2_-VASc score (acronym of Chronic heart failure, Hypertension, Age≥75 years, Diabetes, transient ischemic attack or Stroke, Vascular disease, Age≥65 years, and female sex). TIA: transient ischemic attack, ACE: angiotensin II converting enzyme. Data represent the mean±SD or frequency.

### Holter ECG analysis

**Fig 1:**
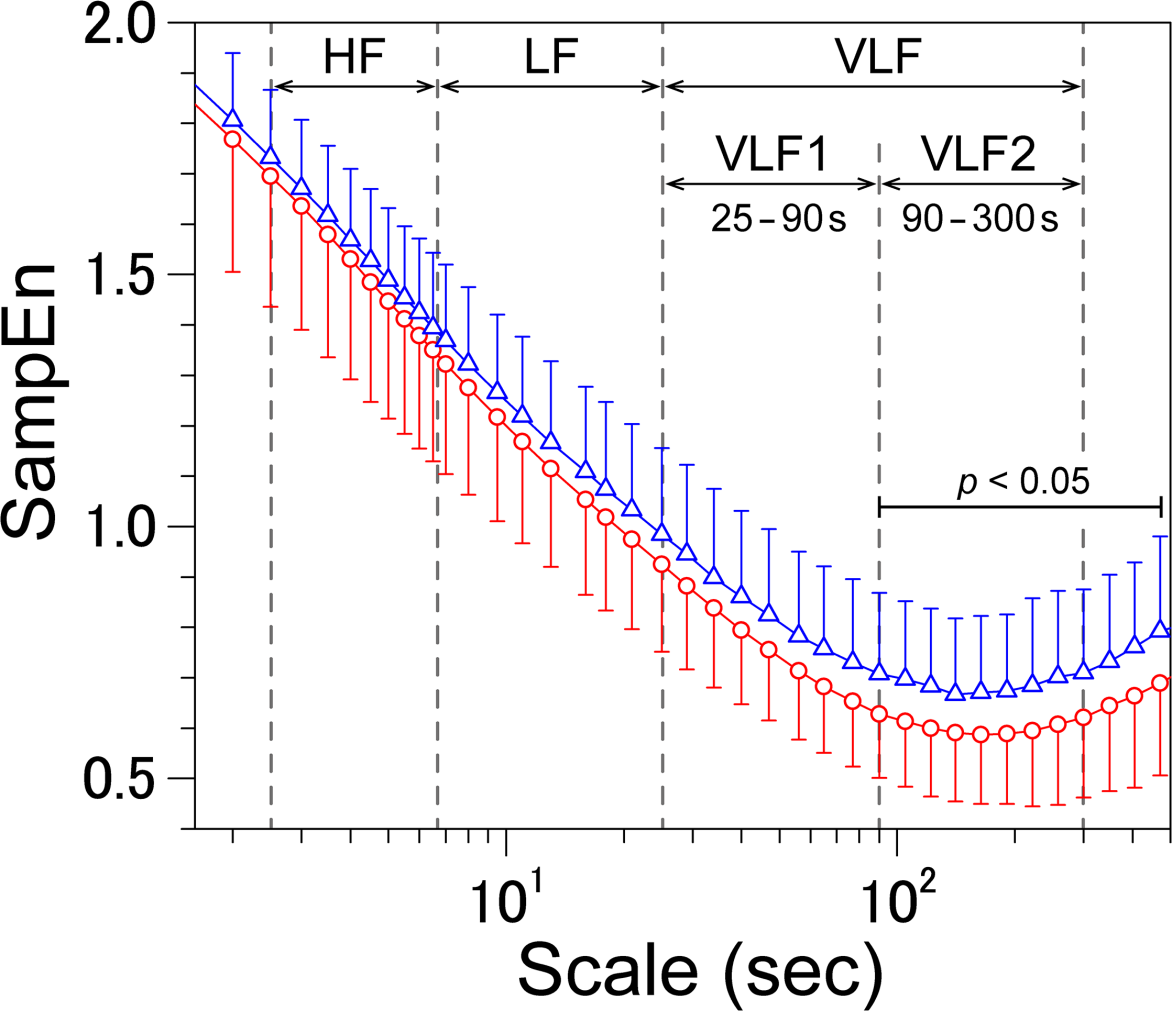
Multiscale entropy profiles of the ventricular response interval time series. Comparison between the groups that developed (blue triangle) and did not develop (red triangle) ischemic strokes during the observation period. Significant differences in the SampEn were observed in the VLF2 range. VRI: ventricular response interval, VLF: very low frequency, SampEn: sample entropy. The error bars represent the SD.

**Fig 2:**
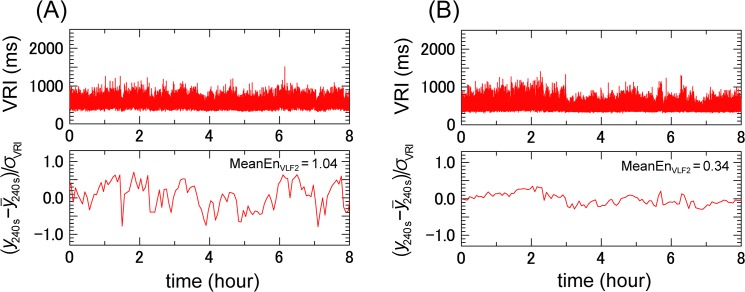
Ventricular response interval and its coarse-grained time series in patients with and without ischemic strokes. (A) A 75-year-old male had an ischemic stroke at 2 months after the recording. (B) A 45-year-old male who did not experience an ischemic stroke during 95 months after the recording. The VRI (upper panel) and its coarse-grained time series with a 240 sec (4 min) scale (lower panel) are shown. The coarse-grained time series is rescaled by subtracting its mean and dividing the differences by the SD (δ) of the VRI time series. VRI: ventricular response interval, MeanEn_VLF2_: mean value of sample entropy of the very-low frequency range 2 (90–300 s).

Fig 1 shows the SampEn of the VRI series of the patients who developed and did not develop ischemic strokes during the observation period. The MeanEn_VLF2_ was significantly greater in those who went on to experience a stroke. Fig 2 shows the representative VRIs and their coarse-grained time series from two patients. A patient who developed an ischemic stroke during the follow up had greater values of the mean value of sample entropy of the VLF2 range (MeanEn_VLF2_) and larger fluctuations (Fig 2A), while a patient that did not experience an ischemic stroke had a lower value of the MeanEn_VLF2_ and smaller fluctuations (Fig 2B).

Table 2 summarizes the HRV parameters. Patients who developed ischemic strokes had higher values of the MeanEn_VLF_ (0.76±0.15 vs. 0.68±0.13, p = 0.02), MeanEn_VLF1_ (0.83±0.16 vs. 0.76±0.14, p = 0.04), and MeanEn_VLF2_ (0.68±0.15vs. 0.60±0.14, p<0.01) than those that did not develop ischemic strokes. In the entire patient population, the MeanEn_VLF2_ had no substantial correlation to the conventional time- and frequency-domain measures (Table 3). A multiple regression analysis demonstrated that the MeanEn_VLF2_ had a weak association with the sex, but had no association with the comorbidities and concomitant drug therapies (S1 Table).

**Table 2:** Holter ECG analysis and heart rate variability measurements.

	Ischemic stroke (n = 22)	No ischemic stroke (n = 151)	P-value
Mean VRI, ms	816±152	811±175	0.91
nVE, beats/24h	1319±4786	1774±5638	0.72
SDVRI, ms	221±57	230±73	0.59
SDAVRI, ms	119±48	115±55	0.70
ULF, ln (ms^2^)	9.23±0.81	9.27±1.01	0.87
VLF, ln (ms^2^)	8.24±0.76	8.28±0.86	0.83
LF, ln (ms^2^)	9.12±0.77	9.20±0.89	0.70
HF, ln (ms^2^)	9.63±0.64	9.72±0.75	0.63
Mean En_HF_	1.57±0.14	1.52±0.24	0.36
Mean En_LF_	1.18±0.16	1.13±0.19	0.21
Mean En_VLF_	0.76±0.15	0.68±0.13	0.02
Mean En_VLF1_	0.83±0.16	0.76±0.14	0.04
Mean En_VLF2_	0.68±0.15	0.60±0.14	<0.01
Slope En_HF_	–0.82±0.17	–0.84±0.18	0.68
Slope En_LF_	–0.71±0.17	–0.73±0.20	0.77
Slope En_VLF_	–0.26±0.16	–0.29±0.18	0.42
Slope En_VLF1_	–0.51±0.14	–0.54±0.21	0.52
Slope En_VLF2_	0.02±0.20	–0.02±0.18	0.43
Scattering index	381±89	390±117	0.66

VRI: ventricular response interval, nVE: number of ventricular ectopy, SDVRI: SD of VRI, SDAVRI: SD of 5-min average VRI, ULF: ultra-low frequency (≥333 s) power, VLF1: very-low frequency (25–90 s) power, VLF2: very-low frequency (90–300 s) power, LF: low frequency (6.7–25s) power, HF: high frequency (2.5–6.7s) power, En: sampling entropy. En_VLF2_: sampling entropy of a very-low frequency range (90–300 s). ln: natural logarithm. Data represent the means±SD.

**Table 3:** Relationships between the clinical features and VRI measures.

	Mean VRI	SDVRI	SDAVRI	ULF	VLF	LF	HF	Mean En_VLF2_	Scattering index
SDVRI	0.73 ^§^								
SDAVRI	0.45 ^§^	0.79 ^§^							
ULF	0.46 ^§^	0.79 ^§^	0.90 ^§^						
VLF	0.71 ^§^	0.89 ^§^	0.62 ^§^	0.72 ^§^					
LF	0.76 ^§^	0.93 ^§^	0.65 ^§^	0.68 ^§^	0.90 ^§^				
HF	0.74 ^§^	0.90 ^§^	0.62 ^§^	0.64 ^§^	0.83 ^§^	0.97 ^§^			
Mean En_VLF2_	0.25 ^§^	0.12	0.14	0.16 *	0.23 ^†^	0.15 *	0.14		
Scattering index	0.78^§^	0.93^§^	0.65^§^	0.67^§^	0.83^§^	0.90^§^	0.90^§^	0.28^§^	
Age	–0.02	–0.12	–0.18 *	–0.18 *	–0.13	–0.08	–0.04	0.01	-0.08
Male sex	0.08	0.20 ^†^	0.23 ^†^	0.22 *	0.25 ^†^	0.18 *	0.20 *	0.16	0.03
Ejection fraction	0.23*	0.25*	0.17	0.13	0.22*	0.25*	0.20	0.08	0.27^§^
Left atrial dimension	0.11	0.05	–0.02	–0.01	0.02	0.07	0.08	-0.04	0.05

Data represent Spearman’s rank correlation coefficients. The abbreviations are as in Tables 1 and 2.

* P<0.05

† P<0.01

§ P<0.001.

### Diurnal variation in the sample entropy

**Fig 3:**
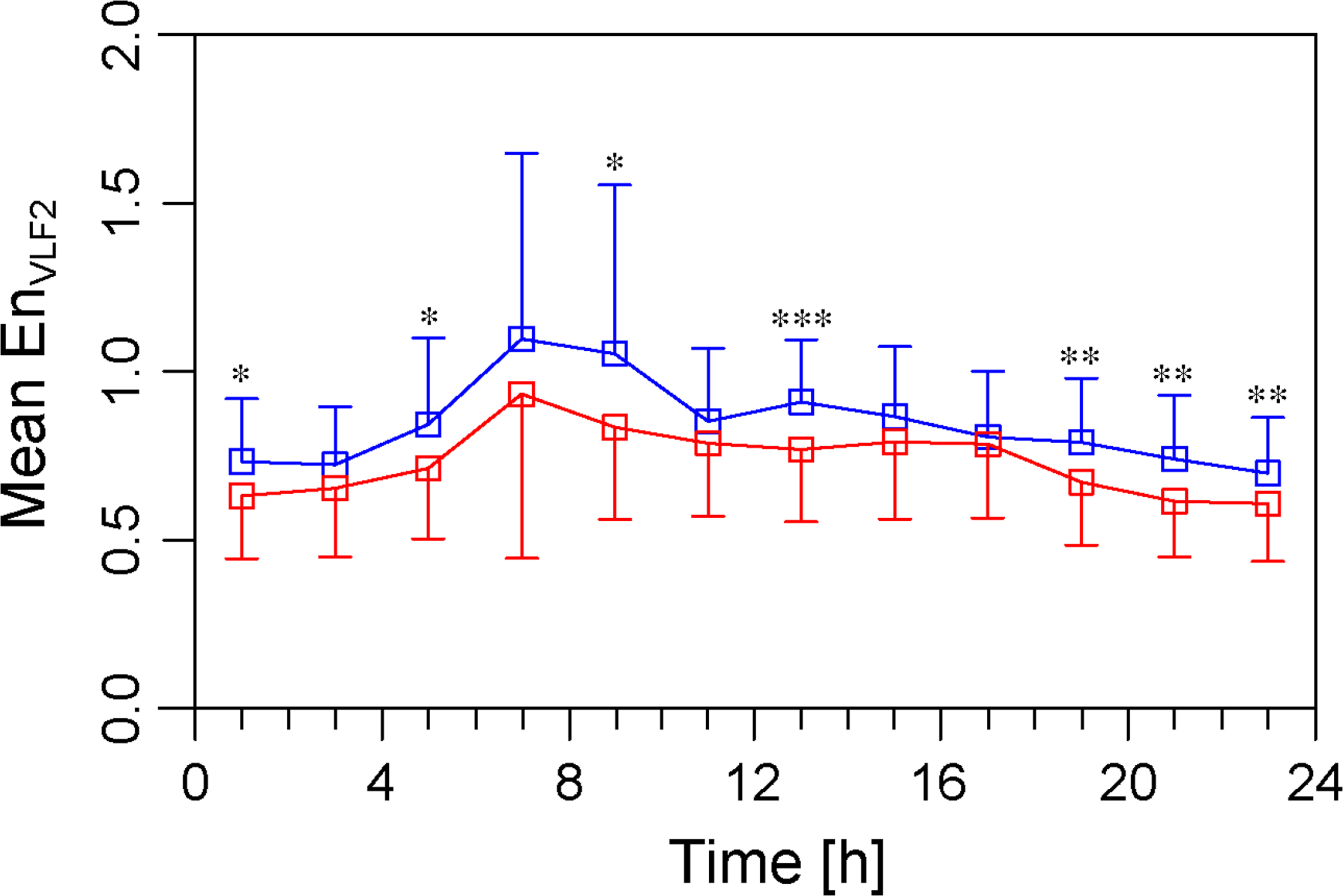
Diurnal variation in the sample entropy in the VLF2 subrange. The MeanEn_VLF2_ for a 4-hour period was plotted every 2 hours, where the center of the interval was assigned as the time. The blue rectangles denote the patients with incident ischemic strokes and the red rectangles denote the patients that did not develop ischemic strokes. MeanEn_VLF2_: mean value of sample entropy of the very-low frequency range 2 (90–300 s).

The diurnal variation in the MeanEn_VLF2_ is presented in Fig 3. There was a significant diurnal variation in the MeanEn_VLF2_ in the study patients (one-way ANOVA, P<0.001). A post-hoc analysis showed that the MeanEn_VLF2_ in those that developed ischemic strokes was greater than that in patients that did not develop ischemic strokes during night (19:00, 21:00, 23:00 and 1:00), early morning (5:00 and 9:00), and at 13:00.

### Comparison of the predictive performance

The C-statistics in the patients with and without receiving antithrombotic drugs are summarized in Table 4. There was no significant difference in the C-statistic between the CHA_2_DS_2_-VASc score and MeanEn_VLF2_ in all patients, but the C-statistic of the MeanEn_VLF2_ was greater than that of the CHA_2_DS_2_-VASc score in the patients that did not receive antithrombotic drugs. Three out of 17 patients with a CHA_2_DS_2_-VASc score of 0 or 1 had an ischemic stroke. The sensitivity and specificity of the mean value of the MeanEn_VLF2_ (0.67) was modest at 66.7% and 64.3%, respectively. The 4-fold cross-validation showed that the sensitivity and specificity of the CHA_2_DS_2_-VASc score was 46% and 56%, and MeanEn_VLF2_ was 59% and 72%, respectively.

**Table 4:** C-statistics.

	CHA_2_DS_2_-VASc	MeanEn_VLF2_	P-value
All patients (n = 173)	0.56 (0.43–0.69)	0.66 (0.53–0.79)	0.07
No antithrombotic treatment(n = 64)	0.54 (0.35–0.73)	0.75 (0.58–0.93)	<0.05
Antithrombotic treatment(n = 109)	0.68 (0.53–0.83)	0.59 (0.39–0.79)	0.31

Abbreviations are as in Tables 1 and 2.

### Time to the event analysis

**Fig 4:**
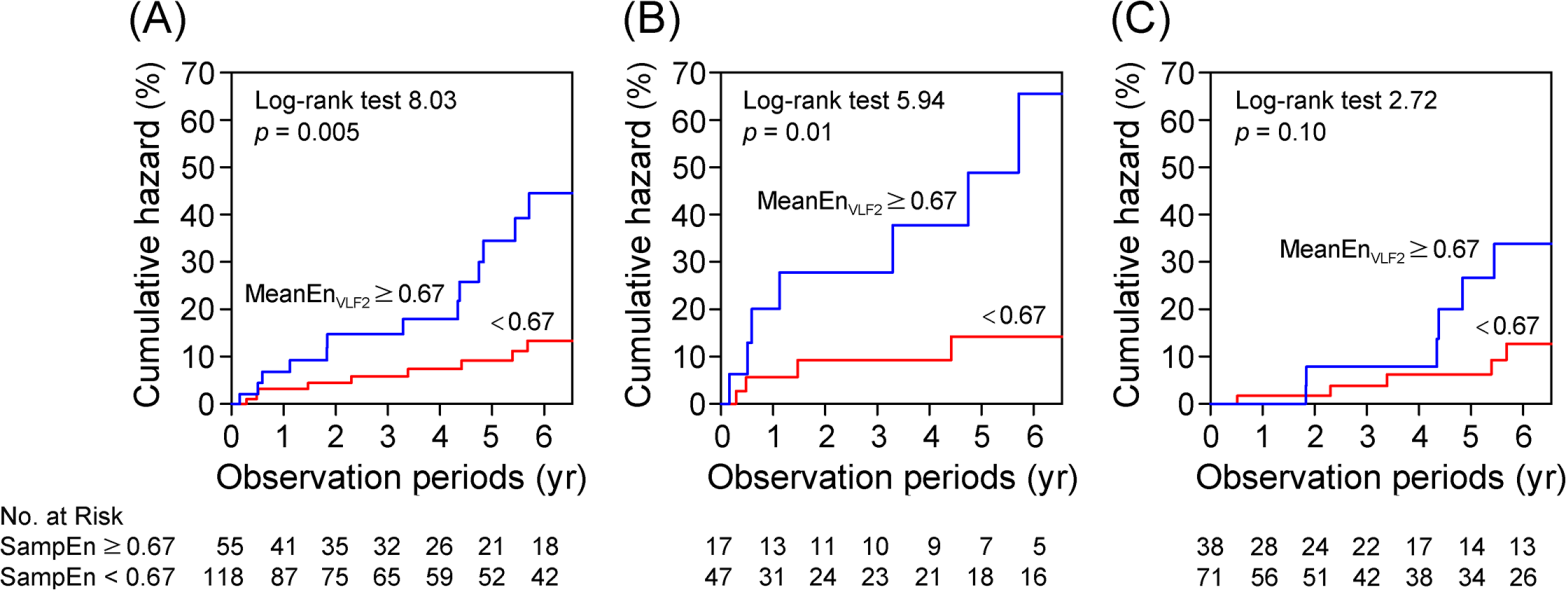
Nelson-Aalen estimate of the ischemic strokes. (**A**) All patients (n = 173), (B) Patients not taking antithrombotic drugs (n = 64), and (C) Patients taking antithrombotic drugs (n = 109).

During the mean follow-up of 47±35 months (median 48 months), ischemic strokes were observed in 22 patients for an annualized event rate of 5.8%. The hazard ratios in the study patients are summarized in Table 5. The adjusted Cox hazard regression analyses revealed that in the entire study population, the MeanEn_VLF2_ was the best independent predictor of an ischemic stroke (HR 1.80, 95%CI 1.17–2.07) followed by the MeanEn_VLF_, MeanEn_VLF1_, and MeanEn_LF_. In patients who did not take antithrombotic drugs, the MeanEn_VLF2_ was the best independent predictor of an ischemic stroke (HR 2.06, 95%CI 1.56–3.48). Nelson-Aalen estimate curves of the patients dichotomized by the mean value of the MeanEn_VLF2_ are shown in Fig 4. There was a significant difference in the ischemic stroke rates in all patients and the patients not receiving antithrombotic drugs.

**Table 5:** Cox proportional hazards regression analysis.

	All patients (n = 173)		No antithrombotic drug ^a^(n = 64)		Antithrombotic drug ^b^(n = 109)	
	HR (95%CI)	P-value	HR (95%CI)	P-value	HR (95%CI)	P-value
Mean En_HF_	1.56 (0.96–2.69)	0.07	1.56 (0.73–3.83)	0.27	1.47 (0.81–2.95)	0.21
Mean En_LF_	1.58* (1.00–2.56)	0.04	1.10 (0.75–1.63)	0.32	1.68 (0.94–3.11)	0.08
Mean En_VLF_	1.74* (1.14–2.62)	0.01	1.99 (1.04–3.67)	0.03	1.65 (0.92–2.95)	0.09
Mean En_VLF1_	1.61* (1.06–2.44)	0.02	1.69 (0.83–3.59)	0.15	1.66 (0.96–2.85)	0.051
Mean En_VLF2_	1.80* (1.17–2.07)	<0.01	2.06* (1.56–3.48)	0.01	1.58 (0.83–3.08)	0.16
Slope En_HF_	1.34 (0.10–9.47)	0.80	0.73 (0.28–1.57)	0.45	1.31 (0.80–1.80)	0.22
Slope En_LF_	0.57 (0.04–6.72)	0.66	0.90 (0.36–2.04)	0.81	0.94 (0.51–1.81)	0.93
Slope En_VLF_	1.05 (0.06–1.50)	0.80	1.83(0.89–4.21)	0.10	0.62 (0.31–1.26)	0.18
Slope En_VLF1_	0.99 (0.64–1.58)	0.99	1.29 (0.69–2.58)	0.42	0.71 (0.36–1.43)	0.33
Slope En_VLF2_	2.70 (0.89–88.8)	0.57	2.16* (1.03–4.84)	0.04	0.64 (0.33–1.25)	0.19

a: adjusted by age and the CHA_2_DS_2_-VASc score in patients who did not receive antithrombotic drugs.

b: adjusted by age, CHA_2_DS_2_-VASc score and antithrombotic drugs (either warfarin or antiplatelet drug).

* denotes P<0.05. HR = hazard ratio per 1-SD increment. CI: confidence interval. The other abbreviations are as in Tables 1 and 2.

## Discussion

To the best of our knowledge, this is the first study to examine the predictive value of the novel mathematical method, MSE, for ischemic strokes in patients with permanent AF. Although the conventional HRV failed to predict the ischemic stroke onset, we found that an increased value of the MSE in the very-low frequency subrange (MeanEn_VLF2_) was an independent predictor of an ischemic stroke. This parameter may be a useful risk stratification measure of ischemic strokes in permanent AF patients with an automated analysis of the Holter ECG.

The CHADS_2_ score [17] has been used to identify patients with AF at risk for a stroke and systemic embolisms. However, it has limited clinical utility because it is unable to stratify patients at truly low risk of a stroke, who may safely avoid anticoagulation. The CHA_2_DS_2_-VASc score was developed to overcome such limitations by including various risk factors and can better distinguish patients between a low and intermediate risk for a stroke than the CHADS_2_ score [4, 18]. In this study we showed that the MeanEn_VLF2_ was an independent predictor of an ischemic stroke after an adjustment for the confounders. Further, our strength was that the MeanEn_VLF2_ was a useful risk stratification measure of ischemic strokes particularly in patients without taking any antithrombotic drugs. We also showed that the MeanEn_VLF2_ had a modest risk stratification power in the patients with a CHA_2_DS_2_-VASc score of 0 and 1.

For sinus rhythm, VRI oscillations in the VLF range are modulated by parasympathetic activity and the endocrine system [19], with a greater power in this frequency range associated with greater parasympathetic activity and a better outcome in patients with cardiovascular diseases [20]. A study of the VRI spectrum in healthy individuals in sinus rhythm and in patients with permanent AF found that the former displayed a 1/*f* slope over the entire spectrum, while that of the latter displayed a 1/*f* slope only in the low frequency range (below a frequency corresponding to a period of approximately 200 s) [21]. Above that frequency, the permanent AF patients exhibited a flat white noise like power. We surmised that the similarity of the power spectrum of AF and sinus rhythm in the VLF range permits the power in the VLF in AF patients to be interpreted as reflecting parasympathetic activity as has been shown for sinus rhythm.

In the previous MSE analysis for permanent AF [8], the MSE profile had been estimated only for a relatively short scale range (<20 beats), corresponding to the HF range and LF range in the conventional HRV analysis. In that short scale range, however, the MSE profile of AF patients only showed a white noise-like behavior as stated above, and suggests that the VRI has no temporal correlations in AF, unlike sinus rhythm. However, by calculating the MSE profile over a wider range of scales that included the VLF range, we were able to confirm the presence of long time scale VRI correlations in AF [21] and quantify them to differentiate patients who developed ischemic strokes from those who did not. This technique demonstrated that AF patients who later developed ischemic strokes displayed higher entropy values.

In this study we found that the MeanEn_VLF2_ had a weak but significant positive relationship with the scattering index, suggesting that the MeanEn_VLF2_ is likely to be correlated with a higher vagal tone [14, 15]. The electrical remodeling in AF may shorten the atrial refractoriness, thereby increasing the number and frequency of fibrillatory waves, which could increase the MSE by enhancing concealed conduction within the AV node [22, 23]. Also, shown in Fig 2, the large baseline drift that appeared in the patients with strokes meant a clustered appearance of relatively long VRIs, which then created more episodes of longer durations of the blood remaining in the atrium, which may have accelerated the thrombus formation in permanent AF, but this hypothesis deserves further study [24].

There is a diurnal variation in the onset of strokes with the greatest prevalence during sleep and the early morning hours [25, 26]. A similar circadian variability is observed in likely thrombogenic precipitants including the blood pressure, vasoconstrictor tone, blood viscosity, and platelet aggregability [27–30]. In this study we observed that patients who developed ischemic strokes exhibited an increased MeanEn_VLF2_ compared to those that did not develop ischemic strokes during the night and early morning hours. Also, we observed postprandial elevation in the MeanEn_VLF2_ (9:00, 13:00, and 19:00). There was no significant difference in the prevalence of diabetes between two groups, but postprandial elevation in MeanEn_VLF2_ may associate with impaired postprandial autonomic nervous system responsiveness. Demonstrations of these associations do not establish a cause-effect relation, but an increased MeanEn_VLF2_ may provide insight into the mechanisms precipitating ischemic strokes in AF.

### Study limitations

This study was an observational, small cohort of Japanese patients at a single institution, which may constitute a selection bias. The presence of diabetic patients or medications may have influenced the activity of the autonomic nervous system. Other important limitations were the underuse of warfarin (only 53% of patients with a CHA_2_DS_2_-VASc score ≥1) and the work was probably underpowered for defining the correlation between the MeanEn_VLF2_ and ischemic strokes in anticoagulated patients. Moreover, the international normalized ratio values in the patients that were anticoagulated who experienced ischemic strokes have not been reported. The differences observed between the groups in the MSE parameters were not large and this may limit the clinical value of the result. Although the findings have internal validity in this cohort, further research is needed in larger samples to determine the external validity of our findings.

## Conclusion

Patients with AF exhibit largely diverse disease characteristics and continue to be at high risk for strokes. The MeanEn_VLF2_ may be a useful marker for prediction of ischemic strokes in permanent AF patients with an automated analysis of the Holter ECG.

## Supporting Information

S1 Data Data set file. (XLSX)

S1 Table Multiple regression analyses of the MeanEn_VLF2. R^2^ = 0.28. En: sampling entropy, VLF2: very-low frequency (90–300 s) power, TIA: transient ischemic attack, ACE: angiotensin II converting enzyme.
(DOC)

## References

[1] Fuster V, Ryden LE, Cannom DS, Crijns HJ, Curtis AB, Ellenbogen KA, et al ACC/AHA/ESC 2006 guidelines for the management of patients with atrial fibrillation—Executive summary: A report of the American College of Cardiology/American Heart Association Task Force on practice guidelines and the European Society of Cardiology Committee for practice guidelines (Writing committee to revise the 2001 guidelines for the management of patients with atrial fibrillation). Circulation. 2006;114(7):700–52.

[2] Camm AJ, Lip GY, De Caterina R, Savelieva I, Atar D, Hohnloser SH, et al 2012 focused update of the ESC Guidelines for the management of atrial fibrillation: an update of the 2010 ESC Guidelines for the management of atrial fibrillation. Developed with the special contribution of the European Heart Rhythm Association. Eur Heart J. 2012;33(21):2719–47. 10.1093/eurheartj/ehs253 22922413

[3] Lip GY, Nieuwlaat R, Pisters R, Lane DA, Crijns HJ. Refining clinical risk stratification for predicting stroke and thromboembolism in atrial fibrillation using a novel risk factor-based approach: the euro heart survey on atrial fibrillation. Chest. 2010;137(2):263–72. 10.1378/chest.09-1584 19762550

[4] Lip GY, Frison L, Halperin JL, Lane DA. Identifying patients at high risk for stroke despite anticoagulation: a comparison of contemporary stroke risk stratification schemes in an anticoagulated atrial fibrillation cohort. Stroke. 2010;41(12):2731–8. 10.1161/STROKEAHA.110.590257 20966417

[5] Heart rate variability: standards of measurement, physiological interpretation and clinical use. Task Force of the European Society of Cardiology and the North American Society of Pacing and Electrophysiology. Circulation. 1996;93(5):1043–65. 8598068

[6] Pincus SM. Approximate entropy as a measure of system complexity. Proc Natl Acad Sci U S A. 1991;88(6):2297–301. 11607165 10.1073/pnas.88.6.2297 PMC51218

[7] Richman JS, Moorman JR. Physiological time-series analysis using approximate entropy and sample entropy. Am J Physiol Heart Circ Physiol. 2000;278(6):H2039–49. 10843903 10.1152/ajpheart.2000.278.6.H2039

[8] Costa M, Goldberger AL, Peng CK. Multiscale entropy analysis of complex physiologic time series. Physical review letters. 2002;89(6):068102 12190613 10.1103/PhysRevLett.89.068102

[9] Costa M, Goldberger AL, Peng CK. Multiscale entropy analysis of biological signals. Physical review E, Statistical, nonlinear, and soft matter physics. 2005;71(2 Pt 1):021906 15783351 10.1103/PhysRevE.71.021906

[10] Yamada A, Hayano J, Sakata S, Okada A, Mukai S, Ohte N, et al Reduced ventricular response irregularity is associated with increased mortality in patients with chronic atrial fibrillation. Circulation. 2000;102(3):300–6. 10899093 10.1161/01.cir.102.3.300

[11] Ho YL, Lin C, Lin YH, Lo MT. The prognostic value of non-linear analysis of heart rate variability in patients with congestive heart failure—a pilot study of multiscale entropy. PLoS ONE. 2011;6(4):e18699 10.1371/journal.pone.0018699 21533258 PMC3076441

[12] Costa M, Goldberger AL, Peng CK. Multiscale entropy to distinguish physiologic and synthetic RR time series. Computers in cardiology. 2002;29:137–40. 14686448

[13] Anan T, Sunagawa K, Araki H, Nakamura M. Arrhythmia analysis by successive RR plotting. J Electrocardiol. 1990;23(3):243–8. 1696610 10.1016/0022-0736(90)90163-v

[14] Hayano J, Sakata S, Okada A, Mukai S, Fujinami T. Circadian rhythms of atrioventricular conduction properties in chronic atrial fibrillation with and without heart failure. J Am Coll Cardiol. 1998;31(1):158–66. 9426035 10.1016/s0735-1097(97)00429-4

[15] Chishaki AS, Sunagawa K, Hayashida K, Sugimachi M, Nakamura M. Identification of the rate-dependent functional refractory period of the atrioventricular node in simulated atrial fibrillation. Am Heart J. 1991;121(3 Pt 1):820–6. 2000749 10.1016/0002-8703(91)90194-m

[16] Schurmann J. Pattern Classification: A Unified View of Statistical and Neural Approaches New York: John Wiley & Sons; 1996.

[17] Go AS, Hylek EM, Chang Y, Phillips KA, Henault LE, Capra AM, et al Anticoagulation therapy for stroke prevention in atrial fibrillation: how well do randomized trials translate into clinical practice? JAMA. 2003;290(20):2685–92. 14645310 10.1001/jama.290.20.2685

[18] Olesen JB, Lip GY, Hansen ML, Hansen PR, Tolstrup JS, Lindhardsen J, et al Validation of risk stratification schemes for predicting stroke and thromboembolism in patients with atrial fibrillation: nationwide cohort study. BMJ. 2011;342:d124 10.1136/bmj.d124 21282258 PMC3031123

[19] Taylor JA, Carr DL, Myers CW, Eckberg DL. Mechanisms underlying very-low-frequency RR-interval oscillations in humans. Circulation. 1998;98(6):547–55. 9714112 10.1161/01.cir.98.6.547

[20] Huikuri HV, Makikallio TH, Peng CK, Goldberger AL, Hintze U, Moller M. Fractal correlation properties of R-R interval dynamics and mortality in patients with depressed left ventricular function after an acute myocardial infarction. Circulation. 2000;101(1):47–53. 10618303 10.1161/01.cir.101.1.47

[21] Hayano J, Yamasaki F, Sakata S, Okada A, Mukai S, Fujinami T. Spectral characteristics of ventricular response to atrial fibrillation. Am J Physiol Heart Circ Physiol. 1997;273(6):H2811–6. 10.1152/ajpheart.1997.273.6.H2811 9435618

[22] Chorro FJ, Kirchhof CJ, Brugada J, Allessie MA. Ventricular response during irregular atrial pacing and atrial fibrillation. Am J Physiol. 1990;259(4 Pt 2):H1015–21. 2171362 10.1152/ajpheart.1990.259.4.H1015

[23] van den Berg MP, Crijns HJ, Haaksma J, Brouwer J, Lie KI. Analysis of vagal effects on ventricular rhythm in patients with atrial fibrillation. Clin Sci (Lond). 1994;86(5):531–5. 8033507 10.1042/cs0860531

[24] Khaykin Y, Wulffhart ZA, Verma A. Left atrial 'sludge' during vagally mediated pause triggered by pulmonary vein antral ablation. Europace. 2011;13(12):1797–8. 10.1093/europace/eur203 21724652

[25] Marshall J. Diurnal variation in occurrence of strokes. Stroke. 1977;8(2):230–1. 557853 10.1161/01.str.8.2.230

[26] Marler JR, Price TR, Clark GL, Muller JE, Robertson T, Mohr JP, et al Morning increase in onset of ischemic stroke. Stroke. 1989;20(4):473–6. 2648651 10.1161/01.str.20.4.473

[27] Ehrly AM, Jung G. Circadian rhythm of human blood viscosity. Biorheology. 1973;10(4):577–83. 4783690 10.3233/bir-1973-10411

[28] Millar-Craig MW, Bishop CN, Raftery EB. Circadian variation of blood-pressure. Lancet. 1978;1(8068):795–7. 85815 10.1016/s0140-6736(78)92998-7

[29] Tofler GH, Brezinski D, Schafer AI, Czeisler CA, Rutherford JD, Willich SN, et al Concurrent morning increase in platelet aggregability and the risk of myocardial infarction and sudden cardiac death. N Engl J Med. 1987;316(24):1514–8. 3587281 10.1056/NEJM198706113162405

[30] Panza JA, Epstein SE, Quyyumi AA. Circadian variation in vascular tone and its relation to alpha-sympathetic vasoconstrictor activity. N Engl J Med. 1991;325(14):986–90. 1886635 10.1056/NEJM199110033251402

