# Supplementary material for: Multiscale Entropy of the Heart Rate Variability for the Prediction of an Ischemic Stroke in Patients with Permanent Atrial Fibrillation
Source: PLoS One. 2015 Sep 1;10(9):e0137144. doi: 10.1371/journal.pone.0137144 (PMC4556684; doi:10.1371/journal.pone.0137144)
Supplement: S1 Table — R2 = 0.28. En: sampling entropy, VLF2: very-low frequency (90–300 s) power, TIA: transient ischemic attack, ACE: angiotensin II converting enzyme. (DOC) [file pone.0137144.s002.doc]

**Supporting information**

**TITLE:** **Multiscale entropy of the heart rate variability improves the predictive accuracy of ischemic strokes in patients with persistent atrial fibrillation**

# **Table S1. Multiple regression analyses of the MeanEn_VLF2**

|  | Regression Coefficient | Standard error | Standard regression coefficient | t-value | P-value |
| --- | --- | --- | --- | --- | --- |
| Intercept | 0.574 | 0.084 | 0.574 | 6.836 | 1.65E-10 |
| Age | 0.001 | 0.001 | 0.084 | 1.004 | 0.317 |
| Sex | 0.051 | 0.023 | 0.172 | 2.189 | 0.030 |
| Congestive heart failure | -0.005 | 0.025 | -0.017 | -0.187 | 0.852 |
| Hypertension | -0.006 | 0.027 | -0.022 | -0.232 | 0.817 |
| Diabetes | -0.046 | 0.037 | -0.097 | -1.250 | 0.213 |
| Stroke or TIA | 0.017 | 0.023 | 0.061 | 0.732 | 0.466 |
| Vascular disease | 0.032 | 0.036 | 0.071 | 0.899 | 0.370 |
| Beta blocker | 0.023 | 0.030 | 0.061 | 0.746 | 0.456 |
| Digitalis | 0.004 | 0.022 | 0.015 | 0.182 | 0.855 |
| Calcium-channel blocker | 0.046 | 0.027 | 0.144 | 1.697 | 0.092 |
| ACE inhibitor | -0.018 | 0.027 | -0.057 | -0.658 | 0.512 |
| Antiplatelet | -0.002 | 0.025 | -0.006 | -0.076 | 0.940 |
| Warfarin | -0.006 | 0.022 | -0.020 | -0.252 | 0.801 |

R^2^=0.28

En: sampling entropy, VLF2: very-low frequency (90–300 s) power, TIA: transient ischemic attack, ACE: angiotensin II converting enzyme.
